# Estimating *In Situ* Zooplankton Non-Predation Mortality in an Oligo-Mesotrophic Lake from Sediment Trap Data: Caveats and Reality Check

**DOI:** 10.1371/journal.pone.0131431

**Published:** 2015-07-06

**Authors:** Olga P. Dubovskaya, Kam W. Tang, Michail I. Gladyshev, Georgiy Kirillin, Zhanna Buseva, Peter Kasprzak, Aleksandr P. Tolomeev, Hans-Peter Grossart

**Affiliations:** 1 Institute of Biophysics, Siberian Branch of the Russian Academy of Sciences, 50/50 Akademgorodok, Krasnoyarsk, 660036, Russia; 2 Siberian Federal University, 79 Svobodny avenue, Krasnoyarsk, 660041, Russia; 3 Department of Biosciences and Centre for Sustainable Aquatic Research (CSAR), Swansea University, Swansea, SA2 8PP, United Kingdom; 4 Department of Ecohydrology, Leibniz-institute of Freshwater Ecology and Inland Fisheries, MU, 310 Ggelseedamm, Berlin, 12587, Germany; 5 Scientific and Practical Center of the National Academy of Sciences of Belarus for Bioresources, 27 Akademicheskaya Street, 220072, Minsk, Belarus; 6 Department of Experimental Limnology, Leibniz-Institute of Freshwater Ecology and Inland Fisheries, 2 Alte Fischerhütte, 16775, Neuglobsow, Germany; 7 Institute for Biochemistry and Biology, PotsdamUniversity, Maulbeeralle 2, 14469, Potsdam, Germany; University of Shiga Prefecture, JAPAN

## Abstract

**Background:**

Mortality is a main driver in zooplankton population biology but it is poorly constrained in models that describe zooplankton population dynamics, food web interactions and nutrient dynamics. Mortality due to non-predation factors is often ignored even though anecdotal evidence of non-predation mass mortality of zooplankton has been reported repeatedly. One way to estimate non-predation mortality rate is to measure the removal rate of carcasses, for which sinking is the primary removal mechanism especially in quiescent shallow water bodies.

**Objectives and Results:**

We used sediment traps to quantify *in situ* carcass sinking velocity and non-predation mortality rate on eight consecutive days in 2013 for the cladoceran *Bosmina longirostris* in the oligo-mesotrophic Lake Stechlin; the outcomes were compared against estimates derived from *in vitro* carcass sinking velocity measurements and an empirical model correcting *in vitro* sinking velocity for turbulence resuspension and microbial decomposition of carcasses. Our results show that the latter two approaches produced unrealistically high mortality rates of 0.58-1.04 d^-1^, whereas the sediment trap approach, when used properly, yielded a mortality rate estimate of 0.015 d^-1^, which is more consistent with concurrent population abundance data and comparable to physiological death rate from the literature.

**Ecological implications:**

Zooplankton carcasses may be exposed to water column microbes for days before entering the benthos; therefore, non-predation mortality affects not only zooplankton population dynamics but also microbial and benthic food webs. This would be particularly important for carbon and nitrogen cycles in systems where recurring mid-summer decline of zooplankton population due to non-predation mortality is observed.

## Introduction

### Importance of non-predation mortality

Zooplankton are the conduit of matter and energy from primary producers to higher trophic levels; consequently population dynamics of zooplankton often determine the functioning of pelagic food webs [1, 2]. Full description of zooplankton population dynamics requires knowledge of growth, reproduction and mortality; of these, mortality is the most poorly constrained [3, 4]. Mortality estimation is commonly derived from imbalance between recruitment rate and observed abundance changes, and the so-estimated mortality rate reflects (but not equals) the sum of predation and non-predation mortalities [5, 6], where non-predation mortality is defined as mortality not due to predation, hence it could have countless possible causes [4]. Predation is often assumed to be the major, if not the only, cause of mortality. In reality, however, non-predation mortality is also important in regulating zooplankton populations [6] and may contribute greatly to the detrital pool in various aquatic systems [7].

A meta-analysis suggested that predation can only account for two-thirds to three-quarters of the total mortality among epipelagic marine copepods [8]. In some lakes and reservoirs, periodic declines of zooplankton populations are caused almost entirely by non-predation factors that leave behind intact carcasses [9–11]. The fate of zooplankton carcasses is to a large extent hinged on their sinking velocity: Slow sinking carcasses provide high quality substrates for microbes and favor nutrient retention within the water column [12], whereas fast sinking carcasses increase the organic matter flux to the benthos [13, 14].

### Measuring non-predation mortality with sediment traps

Non-predation mortality can be estimated on a basis of carcass abundance [9, 15, 16]. For example, Gries and Güde [9] calculated the non-predation mortality of *Daphnia* in Lake Constance as daily loss due to sedimentation of intact and presumed dead individuals into non-poisoned sediment traps to be 0.002–0.18 d^-1^ (0.2% and 18% of the standing stock). Likewise, Frangoulis et al. [16] estimated depth-average non-predation mortality rate of copepods in the Western Mediterranean as <0.01–0.05 day^-1^ by applying the method of Gries and Güde [9] to ‘swimmer-excluding’ sediment traps with all settled animals considered as dead.

An original method of direct *in situ* estimation of non-predatory mortality based on sediment trap data, proposed by Gladyshev and Gubanov [17], was applied to *Daphnia longispina* and *Cyclops vicinus* populations in a small Siberian reservoir (e.g. [11, 18, 19]). The method utilizes the equation of the vertical transport of the carcasses with a source term in the following form:

$$\frac{\partial y}{\partial t}=mN-\frac{\partial F}{\partial z},$$ (1)

where *m* (d^-1^) is the specific non-predation mortality, *F* is the vertical flux of carcasses, *N* and *y* are abundances of live individuals and carcasses (ind m^-3^), respectively. Integration of this equation over the layer 0 < *z* < *h* above the sediment trap yields:

$$\int_{0}^{h}\frac{\partial y}{\partial t}dz=\int_{0}^{h}mNdz+F(0)-F*,$$ (2)

where the vertical flux F* of carcasses at the trap exposure depth *h* (ind m^-2^ d^-1^) is directly measured by sediment traps as

$$F*=\frac{Y}{S}.$$ (3)

Here, *Y* is number of carcasses accumulated in a sediment trap per day (ind d^-1^), *S* is the input area of the trap (m^2^). Using the condition of zero flux of the carcasses across the air-water boundary *F*(0) = 0, and applying the mean value theorem, one arrives at

$$\frac{\partial \bar{y}}{\partial t}+\frac{F*}{h}=\bar{mN},$$ (4)

where the overbar denotes vertical averaging over the layer 0 < *z* < *h* (m). Assuming the mortality rate to be approximately constant within the integration layer, the expression for the non-predatory specific mortality *m* becomes

$$m=\frac{1}{\bar{N}}\frac{\partial \bar{y}}{\partial t}+\frac{F*}{\bar{N}h}.$$ (5)

Sinking velocity of carcasses v* (m d^-1^) at the depth of traps exposure can be found from the concentration of the carcasses at the exposure depth y* (ind m^-3^) and vertical flux F* (Eq 3) as

$$v*=\frac{F*}{y*}=\frac{Y}{S\cdot y*},$$ (6)

Using the definition for the specific rate of elimination of carcasses from the sampling layer, *G* (d^-1^):

$$G=\frac{v*}{h},$$ (7)

and applying forward finite differences for time integration of the differential Eq (5), one arrives at the equation for non-predatory specific mortality:

$$m_{i}=\frac{\Delta y}{\Delta t\cdot N_{i}}+G_{i}\cdot \frac{y_{i}^{*}}{N_{i}},$$ (8)

or, assuming nearly homogeneous vertical distribution of the carcasses (*y*_i_* = *y*_*i*_), as originally proposed by Gladyshev and Gubanov [17]:

$$m_{i}=\frac{\Delta y}{\Delta t\cdot N_{i}}+G_{i}\cdot \frac{y_{i}}{N_{i}}.$$ (8A)

where ∆*y = y*_*i*+1_−*y*_*i*_ is the difference in mean carcass abundances in the layer 0—h (ind m^-3^) over the period ∆*t* = *t*_*i*+1_ − *t*_*i*_ of the trap exposure and zooplankton sampling (day); *i* = 1, 2, … *n*; *n* being the total number of samples taken at the location over the study period.

Eq 8A was originally applied to the shallow (0–2 m), well-mixed layer of small reservoirs (e.g. [11, 17, 19]), while Eq 8 may perform better in deep-water layer studies. Note that, strictly speaking, elimination of carcasses (Eq 7) includes sedimentation, decomposition and ingestion. Sedimentation is however assumed the primary (fastest) component of the elimination because carcasses in general sink faster than they decompose [9, 20–22]. Nonetheless, such an assumption can be properly evaluated by comparing the so-estimated non-predation mortality rate with concurrent population abundance data.

In this study, we applied Eqs 6, 7 and 8A to estimate non-predation mortality rates. Carcass sinking velocity—a key variable in the equations—was obtained by three approaches: a) settling column method, b) settling column method corrected for turbulence resuspension and microbial degradation, and c) *in situ* sediment trap data (Eq 6). Settling column method is commonly used to measure *in vitro* sinking velocity of detrital particles, including zooplankton carcasses, under constant temperature and viscosity in the absence of convective or turbulent motions [23], and sometimes these measurements are extrapolated to *in situ* conditions without sufficient consideration of the effects of turbulence or decomposition (e.g. [16, 24]). Kirillin *et al*. [21] introduced a model to correct settling column measurements for turbulence resuspension and microbial degradation of zooplankton carcasses, but it has not been verified by field observations. Our goal is to compare the mortality estimates by the different approaches, discuss probable causes for discrepancies, and highlight the importance of non-predation zooplankton mortality for food web dynamics and biogeochemical cycling in aquatic systems.

## Materials and Methods

### Study site

Lake Stechlin (53°10' N, 13°02' E), a dimictic oligo-mesotrophic lake in Germany [25], has been intensively studied for the last 4 decades [26]. It is a site member of the Global Lake Ecological Observatory Network (GLEON), and recently its zooplankton live/dead composition [27] and microbial carcass decomposition [12] have been investigated. Our study was conducted on 3^rd^-11^th^ July, 2013 at close to the deepest point (ca. 70 m) of the lake, ca. 100 m from a moored autonomous monitoring station LakeESP. Field permit was granted to the Leibniz institute by the Stechlin natural park authorities on a permanent basis.

### Sediment traps

**Fig 1:**
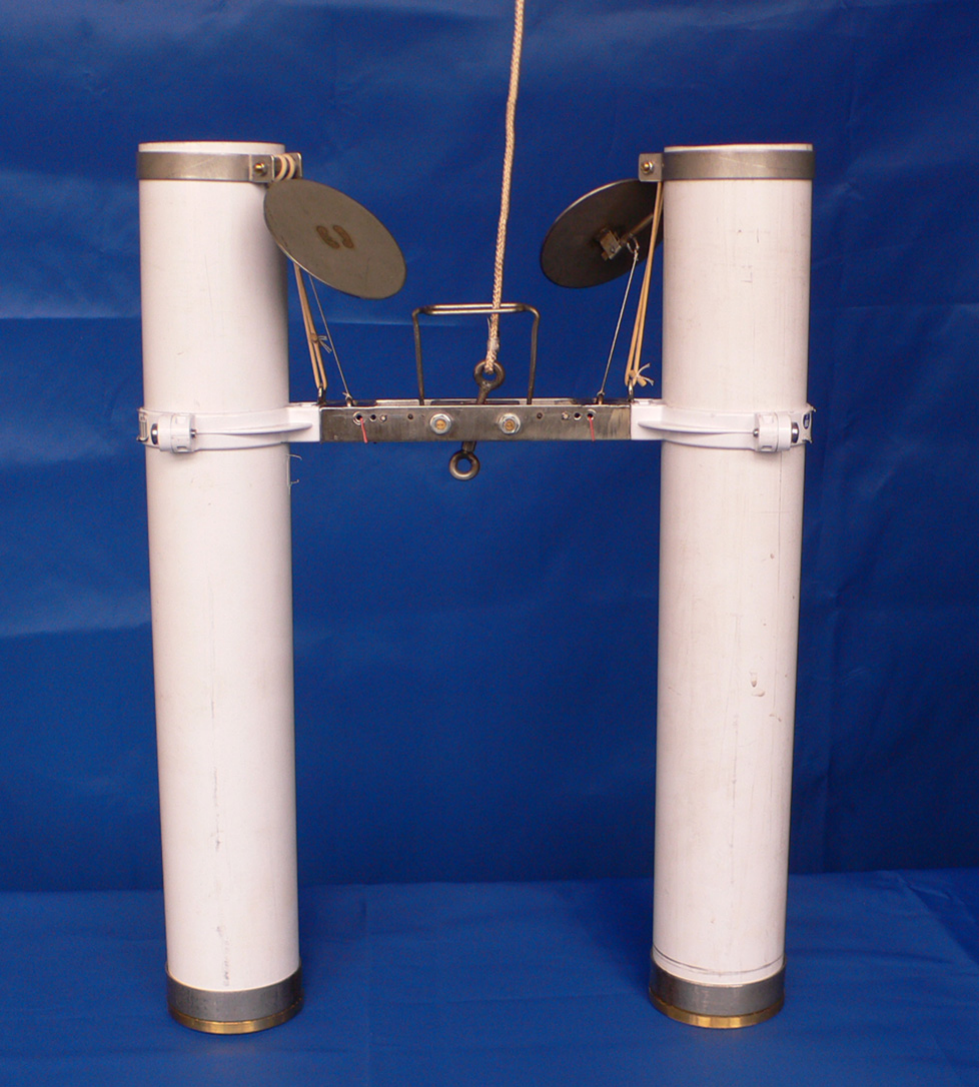
Photo of the sediment trap.

Three pairs of sediment traps were deployed at 12 m (± 1 m) in the lower part of the thermocline each day for 6 consecutive days. Each trap, after the design of Håkanson [28], consisted of a pair of cylindrical collectors with a closing mechanism (Fig 1). Each collector had the dimensions of 0.077 m dia. × 0.485 m height; the height-to-diameter ratio therefore satisfied the recommended value to prevent resuspension [29]. Before deploying, the cylinders were filled with water from 12 m pre-screened through a 90 μm mesh; no poison was used. The cable was anchored, stretched by a submerged buoy and marked by a surface buoy. Trap exposure time was 1 day, except on 10^th^ July when the exposure time was 20 hours. After traps retrieval, zooplankton samples from the paired collectors were pooled, concentrated on a 90 μm mesh and processed to obtain *Y* in Eq 6.

### Water column zooplankton samples

Zooplankton at trap depth were sampled daily at around 10 a.m. using a Schindler-Patalas sampler (volume 14 L, collector mesh size 90 μm) to obtain y* in Eq 6. Samples were pooled from triplicate runs (total 42 L); on 4^th^ July an additional sample was taken after 6 hours of trap exposure. In addition, to obtain depth-averaged *N*_*i*_, *y*_*i*_ and *y*_*i+1*_, pooled samples (total 56 L) were taken at 0, 3, 6 and 9 m on 3^rd^, 5^th^ and 10^th^ July.

### Staining and counting of zooplankton

To distinguish between live and dead zooplankton, samples from the Schindler-Patalas sampler and sediment traps were stained within an hour after collection with aniline blue [27, 30] using a staining device [19, 20], and fixed in 10% formalin. Live (unstained) and dead (stained blue) zooplankton were identified and counted, and their body length measured under a microscope [20]. Entire samples were counted, but for samples with >500 individuals, random subsamples were examined until at least 100 individuals of the same species were counted.

### *In situ* sinking velocity

*In situ* carcass sinking velocity was calculated according to Eq 6. Because samples were pooled from each pair of collectors, trap input area was calculated as: *S* = (0.077/2)^2^π ∙2 = 0.0093 m^2^. y* was calculated as the average of samples taken at the beginning and at the end of the daily trap exposure. For reliable calculations, only the most abundant species in each trap was used, in this case *Bosmina longirostris* (O.F. Müller).

### *In vitro* carcass sinking velocity and carcass density

Experiments were conducted to measure carcass density and *in vitro* carcass sinking velocity. Carcass density was measured by the density gradient method: A saline solution (0.1 g NaCl ml^-1^) was mixed with distilled water in 50-ml centrifuge tubes to create various densities (1.055, 1.060, 1.065, 1.070, 1.075 and 1.080 g ml^-1^). Live *B*. *longirostris* collected from the lake was killed by brief exposure to the saline solution, then briefly rinsed in lake water and examined under the microscope to ensure no air bubbles were trapped underneath the carapace. The carcasses were then gently added individually with a thin pipette into the centrifuge tubes, and their sinking patterns were noted. If the carcass sank or floated quickly, it was removed and transferred to the next higher or lower density. This process continued until the carcass was at or near neutral buoyancy, at which point its density was assumed to be equal to the solution density. If the carcass sank in one solution density but floated in the next higher solution density, its density was assumed to be the median between the two densities.

*In vitro* carcass sinking velocity under constant temperature and absence of turbulence was measured in a 1-L graduated cylinder filled with lake water and equilibrated to room temperature (~20°C) to minimize convection. Fresh carcasses produced as described before were gently released individually just below the surface. The time required for the carcasses to sink at least 20 cm was recorded; if the trajectory clearly deviated from a vertical line the trial was discarded.

### Mortality calculations

Non-predation specific mortality (*m*) was calculated for 0–12 m according to Eq 8A. The specific rate of elimination (*G*) was calculated from Eq 7 using *h* = 12 m and sinking velocity v* determined from Eq 6. Steps were taken to minimize potential errors in estimation of zooplankton and carcass abundances [20]: 1) traps were exposed for ≤24 h to increase accuracy of *Y*; 2) three replicate traps were exposed simultaneously to increase accuracy of *Y* by increasing the total value of *S* and to increase precision of *Y*; 3) large volume plankton samples (42 L) were collected at trap depth to increase accuracy of y*; 4) sampling at trap depth was done 2–3 times per day to increase precision of y* and to integrate daily variations of y*.

### Environmental data

The autonomous environmental station LakeESP measured major meteorological variables (air temperature and humidity, wind characteristics, incoming solar radiation) as well as underwater light conditions, temperature and oxygen profiles. Data were recorded at 10 min interval.

### Turbulence estimation

A free-falling shear microstructure profiler MSS-60 (Wassermesstechnik Prandke) equipped with two airfoil velocity shear sensors was used to measure the dissipation rate of turbulence kinetic energy (*ε*) [31]. The instrument was allowed to fall freely at a speed of 0.5 m s^-1^ taking measurements at 1024 Hz. Series of 10–15 profiles with intervals of 10–15 min were taken at noon on each day. The vertical turbulent diffusivity (*K*_*Z*_) was determined from the shear microstructure profiles [21] and was subsequently used to add turbulence effect to the *in vitro* sinking velocities.

### Carcass sinking and decomposition model

*In vitro* carcass sinking velocity in non-turbulent environment (*U*_*S*_) was estimated as:

$$U_{S}=\frac{BL^{2}}{C_{1}\upsilon +{\left(C_{D}BL^{3}\right)}^{1/2}}$$ (9)

where *L* (m) is the equivalent spherical diameter of the carcass, υ (m^2^ s^-1^) is the kinematic viscosity of water, *B* = *g*∆*ρ/ρ*_*w*_ (m s^-2^) is carcass buoyancy, *g* (m s^-2^) is the gravity acceleration, ∆*ρ* = (*ρ*_*p*_ *−ρ*_*w*_) is the difference between carcass density (*ρ*_*p*_) and water density (*ρ*_*w*_), *C*_1_ = 24 and *C*_*D*_ = 0.75 are empirical constants [32].

The turbulence effect is introduced into the model by adopting the following equation for carcass trajectory in a spatially heterogeneous turbulence field [33]:

$$dZ=-U_{S}dt+\frac{dK_{Z}}{dz}dt+R\sqrt{2K_{Z}dt}$$ (10)

where *Z* is the vertical coordinate of a carcass, *R* is a normally distributed random number with zero mean and variance of 1.

The effect of microbial degradation on carcasses density was parameterized as a function of time and temperature as [34]:

$$\rho_{p}=\rho_{pi}-3.78(1-e^{-0.329T})(\ln(t)+1.369)$$ (11)

where *ρ*_*pi*_ is the initial carcass density (kg m^-3^), *T* is temperature (°C) and *t* is time in hours (*t* = 0 at 0.25 h after death).

### Statistical analysis

Standard deviation (SD), coefficient of variation (CV), standard error (SE) and the Kolmogorov–Smirnov test for normality (D_K-S_) were calculated conventionally using STATISTICA software, 9.0 (StatSoft Inc., Tulsa, OK, U.S.A.). Relative error was calculated as %SE relative to the mean.

## Results

### Environmental conditions, thermal stratification and turbulence

**Fig 2:**
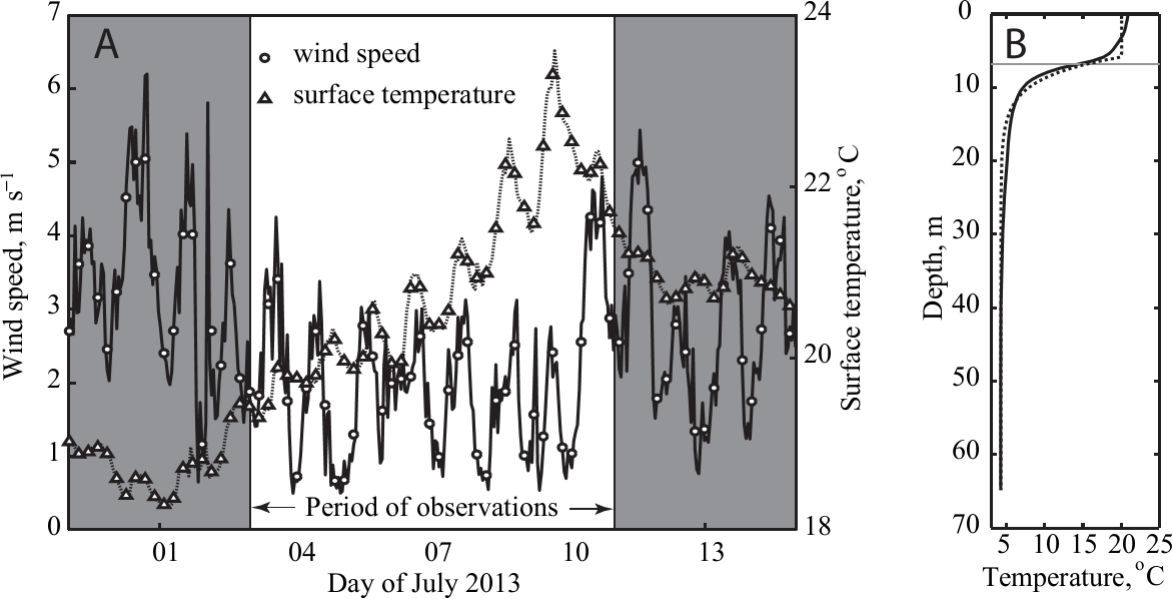
(A) Lake surface temperature and wind speed before, during and after the field experiment. (B) The vertical temperature profile averaged over the observations period. Horizontal dash line marks the bottom of the epilimnion determined from location of the maximum vertical temperature gradient.

**Fig 3:**
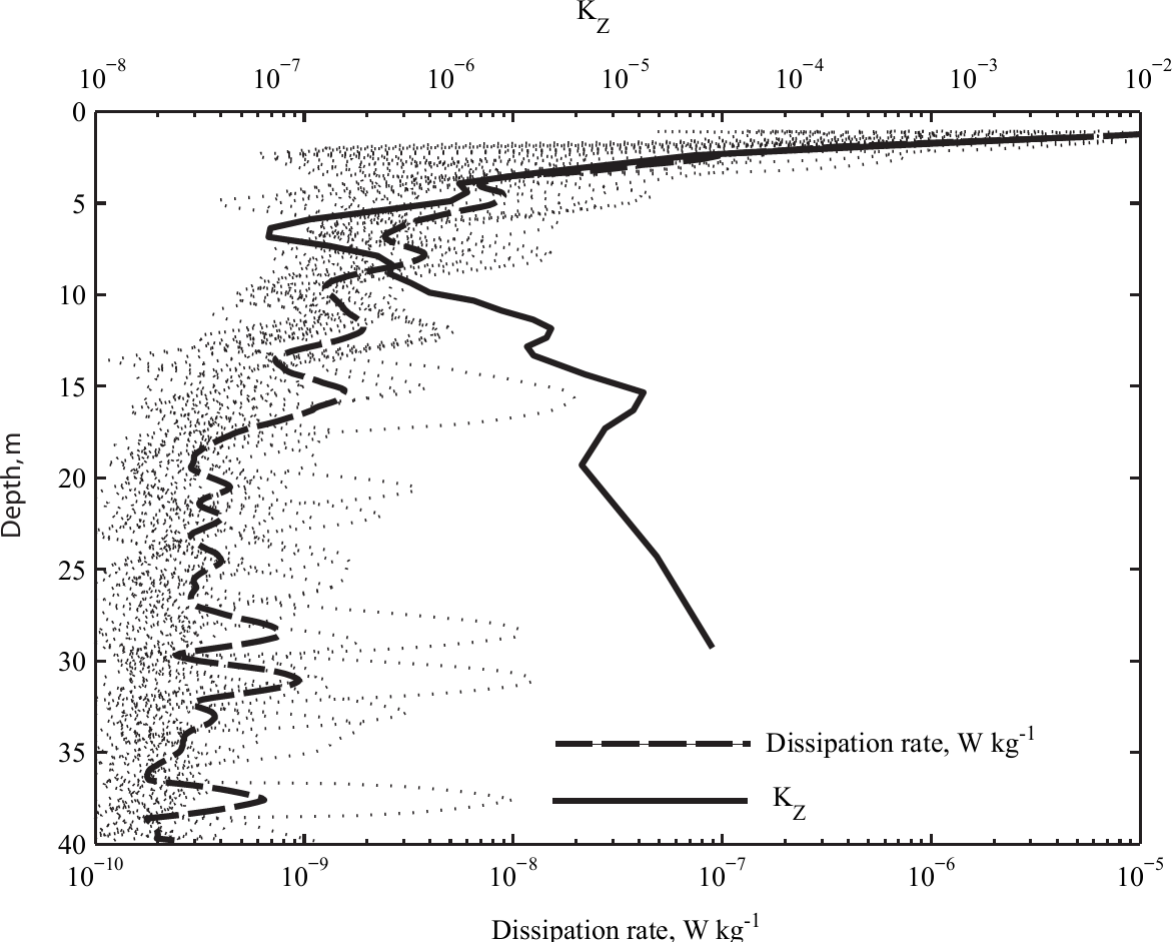
Turbulent mixing within the water column during the field experiment. Turbulent diffusion coefficient *K*_*Z*_ averaged for the study period (thick solid line) and the dissipation rate of the turbulent kinetic energy (thin dash lines are individual profiles; thick dash line is the average for the whole study).

The weather was calm during the study with daily mean wind speed <2 m s^-1^; surface water temperature increased from 19.5°C on 3^rd^ July to 23°C on 10^th^ July (Fig 2A). A strong thermocline began at 8 m, and temperature at the sediment trap depth (12 m) was ca. 7°C (Fig 2B). *ε* decreased from 5×10^−9^ m^2^ s^-3^ at 8 m to 5×10^−10^ m^2^ s^-3^ at 20 m and remained close to the detection limit below (Fig 3). In the epilimnion, *ε* increased rapidly from ~10^−8^ m^2^ s^-3^ at 5 m to ~10^−5^ m^2^ s^-3^ at 1.5 m, characteristic of turbulence produced by wind shear and surface wave breaking [21]. *K*_*Z*_ was close to kinematic molecular viscosity (~10^−6^ m^2^ s^-1^) in the metalimnion, increased slightly in the deeper water, and was high (~10^−4^ m^2^ s^-1^) across the surface mixed layer (Fig 3 **).** Overall, the water column was characterized by a shallow epilimnion exposed to wind mixing, a strong thermocline, and a cold hypolimnion with low mixing intensity between ~15 m and 65 m. Therefore, the bulk of the water column was a nearly homogeneous non-turbulent environment, close to the classical Stokes’ condition.

### Trap and water column samples

Number of *B*. *longirostris* carcasses accumulated in the traps per day varied from 20 to 104 (Table 1), and the data showed a normal distribution (Kolmogorov–Smirnov test D_K-S_ = 0.164, p > 0.20). Relative errors of carcass abundance among the 3 traps were low (12.4–27.6%; Table 1), indicating good trap precision. Carcasses of other zooplankton species were too rare to be included in the analysis. Relative errors of estimation of carcass abundance at trap depth ranged from 1.3 to 66.2% (Table 1). At that depth, dead *B*. *longirostris* comprised 2.4–8.2% of the total abundance, averaging 6.0 ± 0.9% (Table 2). Percentage of dead *B*. *longirostris* was much higher inside the traps averaging 48.0 ± 4.8% (range 37.3–65.1%, estimated only on 9^th^-11^th^ July).

**Table 1:** Number of *Bosmina longirostris* carcasses accumulated in sediment traps per day (*Y* in Eq 6) and carcass abundances at trap depth (12 m, average of 2–3 samples taken at the beginning and at the end of daily trap exposure; y* in Eq 6) in Lake Stechlin.

	Carcasses in sediment trap (ind)					Carcasses at trap depth (ind m^-3^)		
Date in July	Trap 1	Trap 2	Trap 3	CV (%)	Relative error (%)		CV (%)	Relative error (%)
3–4	44	43	20	38.1	22.0	3095	43.5	30.8
4–5	78	48	42	34.4	19.9	2493	18.5	10.7
5–6	63	41	50	21.5	12.4	3056	1.8	1.3
6–7	86	75	52	24.4	14.1	3553	18.2	12.9
9–10	63	103	87	23.9	13.8	6576	93.6	66.2
10–11	35	104	90	47.7	27.6	7965	52.7	37.2
Mean	62.4			39.4	9.3	4087	67.1	22.4

CV = Coefficient of variation.

**Table 2:** Abundance and % dead of *Bosmina longirostris* at trap depth (12 m) in Lake Stechlin.

Date in July	Live (ind m^-3^)	Dead (ind m^-3^)	% dead
3	46190	4048	8.06
4	37857	2143	5.4
4*	nd	2321	nd
5	80167	3016	3.63
6	nd	3095	nd
7	48512	4010	7.6
9	89286	2222	2.4
10	122619	10931	8.18
11	67857	5000	6.86
Mean ± SE	70356 ± 11244	4087 ± 914	6.0 ± 0.9

nd = no data.

*additional sample taken 6 hr after trap exposure.

Live *B*. *longirostris* appeared to congregate in the metalimnion (Table 2). By 10^th^ July, only a few dead individuals remained in the 0–9 m layer, whereas all live ones were found deeper (Table 3). We calculated the mean abundances (*N*_*i*_, *y*_*i*_ and *y*_*i+1*_ for Eq 8A) for the 0–12 m layer by extrapolating the abundances at 12 m to that in the 9–12 m layer, i.e. weighted mean for 0–12 m = [N_(0–9)_×9m + N_12_×(12-9m)]/12m, where N_(0–9)_ and N_12_ are abundances in the 0–9 m layer and at 12 m, respectively (Table 3).

**Table 3:** Abundances and % dead of *Bosmina longirostris* in water column above the traps (0–9 m) and weighted mean for 0–12 m (*N*_*i*_ and *y*_*i*_) in Lake Stechlin.

Date in July	Layer (m)	Live (ind m^-3^)	Dead (ind m^-3^)	*Er* (ind m^-3^)	% dead
3	0–9	20982	2035	748.4	8.84
	0–12	27284	2538	883.2	8.51
5	0–9	23725	520	269.0	2.15
	0–12	37835	1144	485.9	2.95
10	0–9	0	232	146.8	100
	0–12	30655	2907	977.9	8.66

*Er* = Error of carcass abundance estimation (see text for explanation).

**Fig 4:**
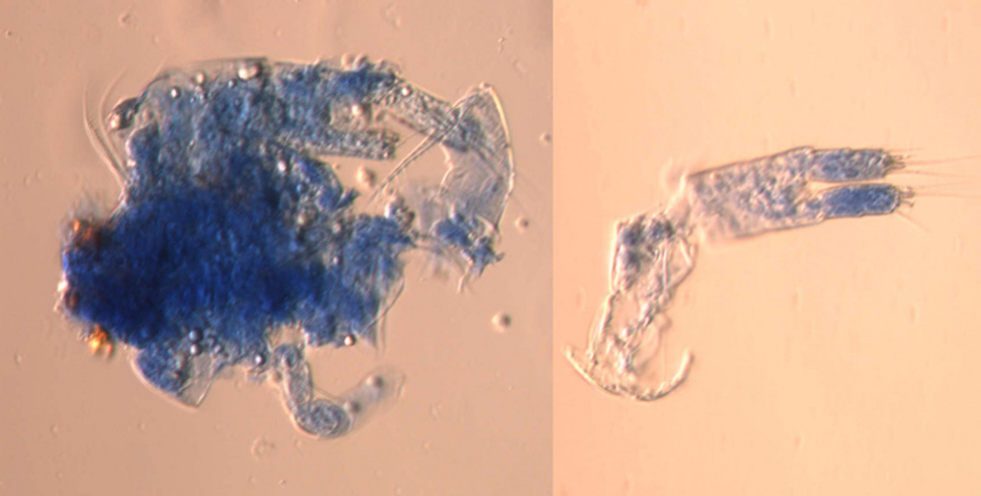
Gut content extracted from *Megacyclops gigas* showing remains of cyclopoid copepods.

Active ‘swimmers’ of the large copepod *Megacyclops (Acanthocyclops) gigas* (Claus) appeared in the traps, which might cause errors in our calculation if they removed some of the carcasses. However, *M*. *gigas* consumes mainly live animals by sucking out the body content of their prey [35], the remains of which would appear very differently than carcasses from non-predation mortality [20]. We did not find sucked carcasses or partial remains of *B*. *longirostris*, and we saw only remains of copepods in extracted gut content of *M*. *gigas* (Fig 4).

### Carcass sinking velocity

*In situ* sinking velocity of *B*. *longirostris* carcasses was calculated for each trap and each day, and it varied from 0.47 to 3.36 m d^-1^ (Table 4). The data were normally distributed (D_K-S_ = 0.116, p > 0.20). The mean ± SD of 10 replicate *in vitro* carcass sinking velocity measurements was 134.26 ± 32.31 m d^-1^ (Table 5). The mean ± SD of 10 replicate measurements of carcass density was 1.070 ± 0.008 g ml^-1^ (Table 6).

**Table 4:** *In situ* sinking velocity of *Bosmina longirostris* carcasses calculated from Eq 6 using data from Table 1 for Lake Stechlin.

	Sinking velocity (m d^-1^)		
Date in July	Trap 1	Trap 2	Trap 3
3–4	1.53	1.49	0.69
4–5	3.36	2.07	1.81
5–6	2.21	1.44	1.76
6–7	2.60	2.27	1.57
9–10	1.23	2.02	1.70
10–11	0.47	1.40	1.21
Mean ± SE	1.71 ± 0.16		

**Table 5:** *In vitro* sinking velocity of *Bosmina longirostris* carcasses measured by settling column method.

Replicate	Sinking velocity (m d^-1^)
1	136.06
2	87.27
3	138.24
4	121.69
5	140.49
6	205.71
7	115.97
8	161.50
9	128.96
10	106.67
Mean ± SE	134.26 ± 10.22

**Table 6:** Density of *Bosmina longirostris* carcasses measured with the density gradient method.

Replicate	Density (g ml^-1^)
1	1.073
2	1.060
3	1.058
4	1.078
5	1.080
6	1.080
7	1.073
8	1.068
9	1.063
10	1.070
Mean ± SE	1.070 ± 0.0025

The equivalent spherical diameter of *B*. *longirostris* was estimated as 243.3 μm by substituting the *in vitro* sinking velocity into Eq 9. This value is close to that determined by microscopy (body length 300 × height 230 × width 120 = 0.008 μm^3^; equivalent spherical diameter ~250 μm). Modeled sinking velocity varied from ~140 m d^-1^ in the epilimnion to ~80 m d^-1^ in the bulk water column. Adding turbulence and decomposition effects decreased the sinking velocity in the hypolimnion to ~75 m d^-1^, which was still much higher than the *in situ* estimate from sediment trap data.

### Mortality rate estimations

Error of carcass abundance estimation (*Er*) can be calculated as *Er* = 2.47 *y* ^0.75^ [36]. This allows us to compare carcass abundances in 0–12 m (*y*_*i*_, Table 3) and in the trap exposure layer (y*, Table 1): on 3^rd^ July, *y*_*i*_ = 2538 ind m^-3^ (*Er* = 883) and y* = 3095 (*Er* = 1025) (Student’s t-test = 0.41, p>0.05); on 10^th^ July, *y*_*i*_ = 2907 (*Er* = 978) and y* = 6576 (*Er* = 1804) (Student’s t-test = 1.79, p>0.05). Thus, mean carcass abundances between the two layers were not significantly different, i.e. *y*_*i*_ ≈ y*, that is a pre-requisite for mortality calculation using Eq 8A. Abundance of live *B*. *longirostris* in 0–12 m (*N*_*i*_ in Eq 8A) was 27284 ind m^-3^ on 3^rd^ July (Table 3); the corresponding *y*_*i*_ was 2538 ind m^-3^ on 3^rd^ July and *y*_*i+1*_ was 2907 ind m^-3^ on 10^th^ July. Thus, ∆*y* = 369 ind m^-3^. *G* = v*/h = 1.71 m d^-1^/12 m = 0.14 d^-1^. *m* calculated using Eq 8A for the interval ∆*t* = 7 days was 0.015 d^-1^. As *y*_*i*_ ≈ y*, this is not significantly different from mortality rate estimated from Eq 8 (*m* = 0.018 d^-1^).

Carcass sinking velocity from settling column measurements was much higher (average *v* = 134.26 m d^-1^), yielding a much higher *m* (1.043 d^-1^). The average *v* from our hydrodynamic model was 75 m d^-1^ and the corresponding *m* was 0.583 d^-1^.

## Discussion

Non-predation mortality has the potential to control zooplankton dynamics and therefore it represents an important factor in population biology and organic matter cycling in aquatic systems. We compared different ways to derive non-predation mortality and evaluate them against observed population abundance data in order to arrive at a more reliable estimate. Carcass sinking velocity of *B*. *longirostris* calculated from our sediment trap data was comparable to that measured by sediment traps (0.70–1.40 m d^-1^) in the small non-stratified reservoir Bugach (Siberia, Russia) [37]. Similar approach has been used to measure sinking velocities of marine snow and fecal pellets [38, 39]. Carcass sinking velocities obtained from lab experiments and model calculations were both much higher, and the almost two orders of magnitude difference led to very different non-predation mortality rates for *B*. *longirostris* in Lake Stechlin: 1.043 d^-1^ (lab), 0.583 d^-1^ (model) and 0.015 d^-1^ (sediment trap). Below we consider probable causes for this discrepancy and the ecological implications of our results.

### Overestimation by settling column method

The settling column method measures *in vitro* carcass sinking velocity under constant temperature and viscosity and in the absence of water motions. Such conditions are rare in natural water bodies; consequently, this method gives the maximum sinking velocities that may not be transferrable to natural conditions [40]. For example, small krill fecal strings in the surface ocean could potentially sink 100–200 m d^-1^ based on settling column measurements, but they were absent in 150 m traps, suggesting that the sinking was greatly decreased by turbulence, stratification or coprophagy [23]. Likewise, the settling column method likely overestimated the *in situ* carcass sinking velocity in our study.

### Possible bias in model calculations

Our hydrodynamic model showed that stratification, turbulence and microbial degradation lowered carcass sinking velocity to ~75 m d^-1^. Applying room temperature in the model produced sinking velocities observed *in vitro*, showing that the model correctly reproduced temperature effects on sinking velocities. It, however, did not account for nighttime convection in the lake. Although the net heat budget for the lake was positive and the average surface temperature increased during our study, air temperatures dropped down to 6°C below the water surface temperature at night, suggesting nighttime surface cooling at a rate of 6–12 W m^-2^. This would produce vertical convective water motions, whose root mean square velocity can be estimated from the surface cooling rate to be ~3–4 mm s^-1^ [41], which is 2–3 times higher than *in vitro* carcass sinking velocity. Hence, nighttime convection might significantly slow down carcass sinking in the epilimnion. In an asymptotic case, if all carcasses were produced in the epilimnion and if carcass sinking was stopped by nighttime convection, the daily mean *in situ* carcass sinking velocity would be decreased by half. Convection will be absent if the atmosphere is warmer than the lake surface. Still, the retention time of carcasses in the epilimnion can be increased by vertical wave currents or Langmuir circulations, resolution of which will require information on fine-scale structure of the velocity fields [21]. Nevertheless, their effects on sinking of carcasses in our study were likely minor given the practically windless conditions during the field experiments.

The Stokes’ equation may overestimate the sinking velocity of irregularly shaped objects. For instance, the sinking velocity of undisturbed marine snow aggregates (2.4–75 mm in length) measured by SCUBA diving was lower than that derived from Stokes’ formula for spherical and ellipsoid objects [42], but similar to those calculated from sediment trap and particle abundance data ([38]; similar to our Eq 6). The modified Stokes formula (Eq 9) takes into account the effect of irregular shape on carcasses sinking that is confirmed by the good agreement of the equivalent spherical diameters provided by Eq 9 and carcasses size determined by microscopy.

Porosity and density of zooplankton carcasses may change due to decomposition and subsequently affect sinking velocity [43]. Zelezinskaya [44] reported that laboratory sinking velocity of *Labidocera brunescens* (Copepoda) carcass one hour after death was 355 m d^-1^, but in 5 hours it decreased to 309 m d^-1^ (-13%) and in 30 hours to 244 m d^-1^ (-31%) under constant salinity and temperature. Carcass sinking velocity of copepodite IV-VI in the York River estuary decreased from 107.1 to 67.4 m d^-1^ in the first 4 hour after death (recalculated from Table 3 in [34]). Sinking velocity of decapod zoea carcasses decreased by 2.2 times in 20 hours and 6.7 times in 70 hours after death [44]. Decomposition of carcasses may also result in formation of interstitial microbubbles of gases, which further increase buoyancy and as a result, carcasses may remain suspended or even float [44]. This “anti-rain” of carcasses may partly explain their high percentage occasionally observed in zooplankton and neuston samples and in surface foam [44, 45].

### Possible bias in trap collection

Appropriate choice of sediment trap dimensions is important for avoiding under-trapping [46]. We used cylinders with a height-to-diameter (H/D) ratio of 6.3, which is sufficient to prevent resuspension even without baffles [47]. According to Lau [48], for cylinder traps with a H/D ratio of 6, Reynolds number needs to be ca. 10 000 to cause resuspension from the trap. Given our trap diameter (7.7 cm) and ambient water temperature (ca. 7°C; corresponding viscosity = 0.0145 cm^2^ s^-1^), such a high Reynolds number would require a current velocity of 18.8 cm s^-1^, much higher than the observed current velocity (5 cm s^-1^) at trap depth. Thus, resuspension from our traps was unlikely.

Another possible error is tilting of the traps [49]. Our trap design maintained the cylinders in upright position independent of cable angle [28]. The traps were moored to a small surface marker buoy and a large subsurface one. Occasionally the subsurface buoys got close to surface causing vibration of the cables and possibly the traps. However, considering the low variability among replicate traps (Table 1) and the calm weather, possible error due to these occasional cable motions was likely negligible.

The coefficients of variance and relative errors of total and carcass abundances were low to moderate (Table 1), which fall within the limits of error of zooplankton abundance estimation of 25–66% for similar sampling method [36]. The one exception was 9-10^th^ July when both live and dead *B*. *longirostris* abundances were increasing. Given the large sample volumes and replications, we believe that we estimated carcass abundances (in water and in traps) with good precision despite known high spatial heterogeneity and patchiness of *Bosmina* spp. [50, 51].

*Bosmina longicornis* has been shown to perform diel vertical migration (DVM) [52]. Even if it occurred in Lake Stechlin, it would not affect *y* or ∆*y* in Eq 8A, nor would it affect our sediment trap data because the traps were not poisoned, they were set out for 20–24 hours, and only carcasses in the traps were used for the calculations. DVM, however, could lead to an underestimation of *N*_*i*_ in Eq 8A and subsequently an overestimation of non-predation mortality rate. Nevertheless, the estimated non-predation mortality rates from Eq 8A were consistent with the water column abundance data, suggesting that any error due to DVM was likely small.

### Potential effects of swimmers

Despite the presence of *M*. *gigas* in the traps, they are not known to feed on carcasses [35] and *Bosmina* (our gut content data) and therefore did not affect our calculations. Nevertheless, if we assume they did, we can estimate the associated errors based on their daily energy expenditure. Krylov [35] estimated a respiration rate of 0.29 μl O_2_ ind^-1^ h^-1^ at 19°C for *M*. *gigas*. Assuming a body wet weight of 0.348 mg for *M*. *gigas* [53] and an ambient temperature of 7°C, we estimated the daily energy expenditure as R = [(0.00029 × 24 × 4.86) / 2.9] / 0.6, where 4.86 cal ml^-1^ O_2_ is oxycalorific coefficient, 2.9 is temperature correction for Q_10_ = 2.3 [54], and 0.6 cal mg^-1^ is caloric content of wet mass [35]. The so-estimated respiratory cost was 0.019 mg wet weight ind^-1^ d^-1^. Assuming an assimilation efficiency of 0.8, the required prey consumption would be 0.024 mg ind^-1^ d^-1^. Given a wet weight of 0.0049 mg for *Bosmina* [53], this translates to a consumption of ~5 carcasses ind^-1^ d^-1^. The average number of *M*. *gigas* in our traps was 41±6 ind, which could consume 205 *Bosmina* carcasses. The corrected carcass abundance in the traps would be ~267 and the *in situ* carcass sinking velocity would increase to 7.0 m d^-1^. The corresponding *m* would be 0.056 d^-1^, which still would not explain the discrepancy with the other two estimates.

### Reality check of non-predation mortality estimates

Based on *in vitro* carcass sinking velocity, we obtained a non-predation mortality rate of 1.04 d^-1^ for *B*. *longirostris*, which is unrealistically high. If we used a sinking velocity of 75 m d^-1^ from model calculations, the mortality rate would be 0.58 d^-1^, and the *B*. *longirostris* population would have completely collapsed in a few days, which obviously was not the case in our study (cf. [11]). The non-predation mortality rate derived from sediment trap data (0.015 d^-1^) is comparable to physiological death rate for marine and freshwater zooplankton (0.01–0.05 d^-1^; [3, 22]). This value also falls within the non-predation mortality rates of *Daphnia* measured by sediment traps in Lake Constance (0.002–0.18 d^-1^; [9]) and of mesozooplankton in coastal Mediterranean measured by ‘swimmer-excluding’ sediment traps (<0.01–0.05 d^-1^; [16]).

At a velocity of 1.71 m d^-1^ a carcass would take ~6 days to sink 12 m, during which time an unknown amount of the carcass materials could be lost to decomposition and detritivory in the upper water column. Although the copepod *M*. *gigas* was unlikely to feed on the carcasses, we could not rule out the possibility that other planktivores (including fish) may have consumed some of the carcasses. For example, *B*. *longirostris* and *B*. *coregoni* are major prey for the fish *Coregonus albula* in the summer in Lake Stechlin [55]. Although it is uncertain whether the fish feed on carcasses, we expect the true non-predation mortality rate to be somewhat higher than 0.015 d^-1^. Elliott and Tang [6] showed that non-predation mortality accounted for 12% of the total *Acartia tonsa* copepodite mortality over 2 years. Following their work, we estimate that the total (predation + non-predation) mortality of *B*. *longirostris* in Lake Stechlin would be ca. 0.13 d^-1^. Assuming a spherical diameter of 0.25 mm, we estimated a biovolume of 0.008 mm^3^ for *B*. *longirostris*. Applying this value to the algorithm of Hansen *et al*. [56] gives us an estimated maximum specific growth rate of ca. 0.24 d^-1^. These crude approximations show that the total mortality rate was on par with the growth rate, meaning that the population size should be rather stable. Judging from Tables 2 and 3, the abundance of live *B*. *longirostris* did not show any consistent increase or decrease, suggesting that the population was at close to equilibrium during our study. Similar findings were reported by Ivanova [54]: seasonal average total mortality rate of *B*. *longirostris* in a Russian lake was 0.13 d^-1^, measured juvenile growth rate 0.20 d^-1^ and generative growth rate of adults 0.23 d^-1^; overall for planktonic crustaceans the average specific production C_B_ could be related to the average specific total mortality rate *m* as C_B_ = (1.86 ±0.60) *m* [54].

### Conclusion and implications for future research

Estimation of non-predation mortality of *B*. *longirostris* in Lake Stechlin was sensitive to carcass sinking velocity, which varied widely among the measurement methods. Mortality rates derived from *in vitro* sinking velocity and its model correction were both unreasonably high; only the mortality rate from *in situ* sediment trap measurements was realistic and consistent with concurrent population abundance data. Nevertheless, the actual mortality rate was expected to be slightly higher than 0.015 d^-1^ because of likely loss of some carcasses within the water column and underestimation of carcass abundance at trap depth, but these errors will be smaller for sediment traps set at shallower depth as long as the ambient current is below the resuspension threshold. Shallower depth would also minimize differences in carcass concentrations within the studied layer (*y*_*i*_) and at the trap depth (y*) because physical and ecological conditions tend to be more homogenous in thinner strata. The thinner the strata being studied, the greater the role of sedimentation in carcass elimination and the greater the accuracy in using Eqs 8 and 8A.

Despite the lack of evidence of ingestion of carcasses by *M*. *gigas*, hypothetical calculations of the swimmers’ consumption in the traps would potentially increase carcass sinking rate and the corresponding mortality rate by ~4 times. Thus, in cases when swimmers occur in traps, their gut contents and feeding biology need to be taken into careful consideration.

As we demonstrated, consecutive days of trap deployment along with water column sampling are needed to perform a reality check of the results; i.e. doing only one or the other and doing snap-shot sampling could give a wrong picture of the population dynamics. To further improve the sediment trap approach, imaging device may be used concurrently to observe and quantify sinking carcasses *in situ* [39, 57].

Our field data suggest that zooplankton carcasses might remain in the water column for days, during which time they could function as microbial hotspots supporting elevated bacterial production and accelerating nutrient recycling in the water column ([22] and reference therein). The labile organics from zooplankton carcasses may even provide a priming effect to facilitate degradation of recalcitrant allocthonous organic matter [58, 59]. In lakes and reservoirs where zooplankton experience recurring mass mortality due to non-predation factors [10, 18, 19], sinking carcasses can also provide a significant pulse of nutrients to the benthos [7, 60]. Therefore, the study of non-predation mortality is relevant to understanding not only zooplankton population dynamics but also biogeochemical cycles and the microbial and benthic food webs.
